# Clinician-led secondary triage in England’s urgent care delivery: a cross-sectional study

**DOI:** 10.3399/BJGP.2022.0374

**Published:** 2023-05-16

**Authors:** Vanashree Sexton, Helen Atherton, Jeremy Dale, Gary Abel

**Affiliations:** Unit of Academic Primary Care, Warwick Medical School, University of Warwick, Coventry.; Unit of Academic Primary Care, Warwick Medical School, University of Warwick, Coventry.; Unit of Academic Primary Care, Warwick Medical School, University of Warwick, Coventry.; University of Exeter Medical School, Exeter.

**Keywords:** cross-sectional studies, digital triage, primary health care, urgent care, National Health Service, British, emergencies

## Abstract

**Background:**

Clinician-led secondary triage, following primary triage by the NHS 111 phone line, is central to England’s urgent care system. However, little is known about how secondary triage influences the urgency attributed to patients’ needs.

**Aim:**

To describe patterns of secondary triage outcomes and call-related factors (such as call length and time of call) associated with upgrading/downgrading of primary triage outcomes.

**Design and setting:**

Cross-sectional analysis of secondary triage call records from four urgent care providers in England using the same digital triage system to support clinicians’ decision making.

**Method:**

Statistical analyses (mixed-effects regression) of approximately 200 000 secondary triage call records were undertaken.

**Results:**

Following secondary triage, 12% of calls were upgraded (including 2% becoming classified as emergencies) from the primary triage urgency. The highest odds of upgrade related to chest pain (odds ratio [OR] 2.68, 95% confidence interval [CI] = 2.34 to 3.07) and breathlessness (OR 1.62, 95% CI = 1.42 to 1.85; reference: abdominal pain) presentations. However, 74% of calls were downgraded; notably, 92% (*n* = 33 394) of calls classified at primary triage as needing clinical attention within 1 h were downgraded. Secondary triage outcomes were associated with operational factors (day/time of call), and most substantially with the clinician conducting triage.

**Conclusion:**

Non-clinician primary triage has significant limitations, highlighting the importance of secondary triage in the English urgent care system. It may miss key symptoms that are subsequently triaged as requiring immediate care, while also being too risk averse for most calls leading to downgrading of urgency. There is unexplained inconsistency between clinicians, despite all using the same digital triage system. Further research is needed to improve the consistency and safety of urgent care triage.

## INTRODUCTION

Urgent care in England is delivered by a range of healthcare providers and is intended to provide care for patients with non-life-threatening illness or injury where urgent attention may be needed.^1^ Digital triage is widely used within urgent care;^2^ it involves use of software-based digital triage tools to generate referral and/or self-care advice based on the patients’ symptoms.

**Figure 1.**
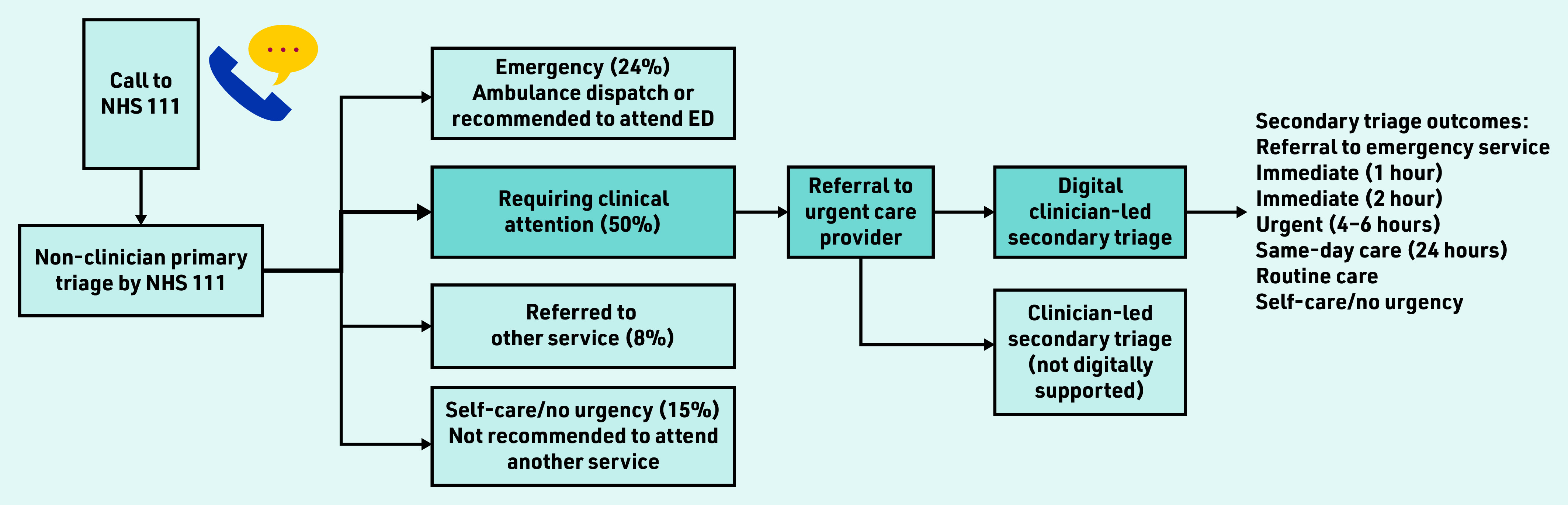
*Referral routes for patients triaged by NHS 111; approximate percentages provided.^5^ Blue referral route represents patients referred to secondary, digitally supported, clinician-led triage, which is the patient population for this study. ED = emergency department.*

In England, the NHS 111 national health advice service provides urgent care advice to patients; the service operates 24/7 and receives >50 000 calls daily.^3^ Patients undergo an initial primary triage, which is conducted by a call operator (a non- clinician, with no clinical training) using the NHS Pathways^4^ digital triage tool. Following primary triage approximately 50% of calls are triaged as requiring urgent clinical attention^5^ and are referred to an urgent care provider for secondary triage (Figure 1).

England’s non-clinician-led model is unlike most other countries where patients are assessed directly by a clinician (any healthcare professional, such as a nurse or GP).^6^ Previous studies have focused on direct clinician triage^7^^–^^13^ with frequent presenting symptoms being abdominal^7^^–^^11^ and respiratory^10^^,^^11^ related, and younger age groups making up higher proportions of calls.^9^^,^^12^ Triage outcome urgency has been described as increasing with age in adults.^8^^,^^10^

To the authors’ knowledge, no previous studies have reported on how triage outcomes change between primary and secondary triage, where call upgrading or downgrading occurs in secondary triage. For example, a call by a non-clinician assigned an urgency level of immediate care within 2 h may be subsequently downgraded to routine care in secondary triage. Such research is needed to enable insight into the safety and effectiveness of two-step triage and whether this approach may expedite or delay patients receiving the care needed. Referral to emergency services should only occur rarely in secondary triage, as such cases should have been referred to emergency services directly from NHS 111. Conversely, if primary triage is too risk averse it may create avoidable workload pressure on both emergency and urgent care providers, and potentially increase delays.

The aim of this study was to:

- explore secondary triage service use;
- understand the factors influencing secondary triage outcome urgency; and
- explore how triage outcome urgency changes between primary and secondary triage.

As the study timing coincided with the COVID-19 pandemic, its impact on utilisation was secondarily considered within the first aim.

**Table:** How this fits in

Two-step triage is well established in England’s urgent care delivery; however, patterns of triage outcomes have not previously been reported. This study is the first, to the authors’ knowledge, to report how triage outcomes change between non- clinician triage and subsequent secondary clinician triage. Although most calls were downgraded in urgency by clinicians, some were upgraded. This study highlights the key presenting symptoms where clinical risk may have been underestimated in non-clinician triage and the clinician variation in secondary triage that requires further research.

## METHOD

### Design and setting

This cross-sectional study focuses on secondary triage, where calls are triaged by clinicians (nurses) using the Odyssey digital triage tool; these calls represent the 50% identified as requiring clinical attention following primary triage. Secondary triage was conducted within four England- based urgent care providers (covering a population of >6 million; these are not the only providers of secondary triage in England). See Supplementary Boxes S1 and S2 for further information about England’s primary and secondary triage, respectively.

### Outcome measures

Key outcomes included the odds of:

- urgent triage outcomes (care within 6 h or less) selected by the clinician in secondary triage; and
- ‘upgrading’ and ‘downgrading’ of primary triage urgency, where the secondary triage outcome selected is more urgent or less urgent, respectively, than urgency assigned in primary triage.

### Dataset

- The anonymised dataset contained secondary triage call records, 1 April 2019 to 1 October 2020, and included patient and call information: anonymised patient number, time/date of call, call length, patient age, sex, Index of Multiple Deprivation (IMD) decile^14^ (derived from patient postcode); presenting symptom (an indicator derived from symptom category attached to the first triage question), and all questions and answers addressed in each call.
- Service provider and anonymised ID of clinician who conducted triage.
- Triage outcomes:
  - primary triage urgency code (dxcode): a preliminary indicator of care recommendation and timeframe (for example, contact primary care service within 6 h) assigned by a non-clinical operator during primary triage, using the NHS Pathways tool (see Supplementary Table S1 for codes); and
  - clinician-selected outcome: the Odyssey triage outcome selected by the clinician in secondary triage.

The ‘clinician-selected’ outcome corresponded to one of seven urgency levels: emergency; immediate care within 1 h; immediate care within 2 h; urgent care within 4–6 h; same-day care within 24 h; routine primary care appointment; and self-care/no urgency (including advice to contact a different service).

### Analysis

For analysis purposes, in this study a binary ‘urgent’ triage outcome was defined for use in regression models; this applied to calls with a triage outcome of clinical care within 6 h or less (including the emergency, <1 h, <2 h, and <4–6 h triage levels in Odyssey); this represents a standard urgent care timeframe in England.^15^

**Figure 2.**
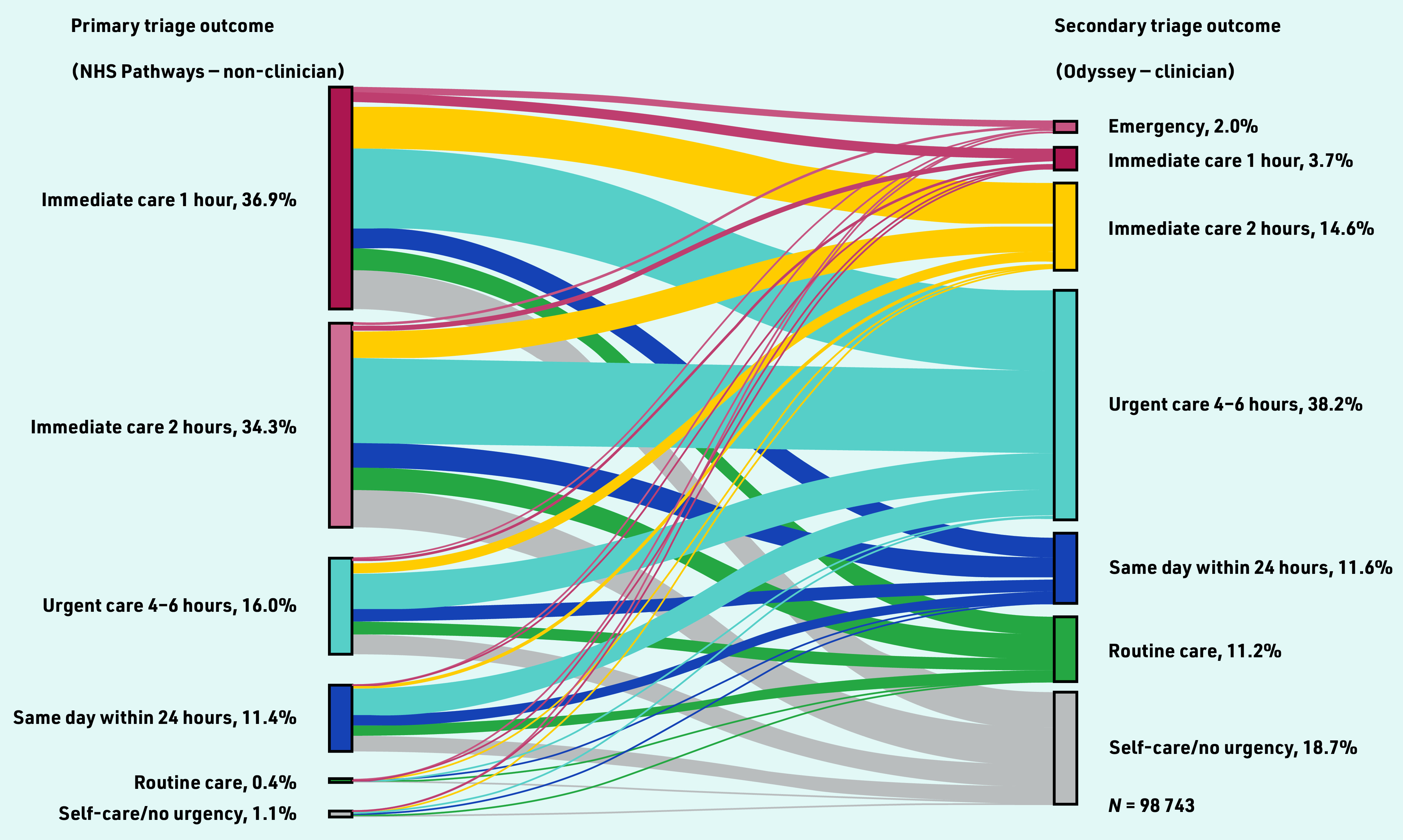
*Sankey visualisation: change in triage outcomes between primary and secondary triage for calls where primary and secondary triage outcome urgencies were available.*

To investigate change between primary and secondary triage urgency, the primary triage urgency code timeframe was mapped to match the urgency levels defined in Odyssey (see Supplementary Table S1); mapping was required to help summarise primary triage outcomes (see Supplementary Table S2) and to enable visual comparison of change between primary and secondary triage outcomes (Figure 2). Mapping was manually conducted with the support of the clinician within the study team. Calls were coded as upgraded or downgraded if the secondary triage urgency was more, or less, urgent than the primary triage urgency. The analysis included three stages.

#### First stage

Descriptive analyses were used to explore call and patient characteristics and primary/secondary triage outcomes. To explore utilisation before and after the start of COVID-19, and to mitigate the impact of seasonal variation, two time periods covering the same months were compared: April–September (2019) and April–September (2020).

#### Second stage

A Poisson regression examined call rates considering local population denominators (age, sex, and deprivation). Population denominator data were created based on clinical commission groups (England-based regions, where the service providers operate) using publicly available datasets.^15^^,^^16^

#### Third stage

Three mixed-effects logistic regression models investigated factors associated with the three key outcomes: a) urgent triage outcomes (care within 6 h or less); b) upgraded calls; and c) downgraded calls.

Models included fixed effects: patient sex, age group, deprivation, and presenting symptom; service provider; day of week; and time of day. Upgrading/downgrading models included additional fixed effects: number of calls triaged by the clinician within the full dataset (an indicator of the clinician’s familiarity with the digital triage tool) and call length. A random intercept for the clinician user conducting the triage was included in all models, enabling the authors to quantify the variability between individual clinicians.^17^

Analyses were conducted using Stata (version 17).

## RESULTS

A total of 289 810 calls about 231 419 individual patients were triaged by 259 different clinicians. Of these, 177 786 calls, across three care providers, had triage questions and answers available (one service provider used an older version of the triage software and was only included in the first two analysis stages). Of the 177 786 calls, 98 946 (56%) had a primary triage urgency code available; remaining calls were excluded: 66 687 (38%) had no code (they did not undergo primary triage as they were referred from a different service) and 12 153 (7%) had >1 code.

### Service utilisation, patient characteristics, and triage outcomes

Proportions of calls were greatest in the youngest age groups (with calls about patients aged <4 years making up around 20% of calls); there were greater proportions of calls about female patients in the most deprived groups (see Supplementary Table S2). These were reflected in call rates, with the highest call rates in the infancy age group (rate ratio [RR] 5.32, 95% confidence interval [CI] = 5.21 to 5.44) and oldest age group (≥85 years) (RR = 4.17, 95% CI = 4.07 to 4.26); reference: 35–44-year-olds; and higher rates in more deprived groups and in female patients. After the start of COVID- 19 the overall call rate decreased by 7% (Table 1).

**Table 1.** Call rates: characteristics of patients by rate ratios from adjusted Poisson regression

**Subgroup**	**Rate ratio (95% CI)^a^**
**Age group**	
<24 months	5.32 (5.21 to 5.44)
2–4 years (young child)	1.91 (1.87 to 1.96)
5–15 years (child)	0.74 (0.72 to 0.75)
16–24 years (young adult)	1.27 (1.24 to 1.30)
25–34 years	1.31 (1.29 to 1.34)
35–44 years	Reference
45–54 years	0.90 (0.88 to 0.92)
55–64 years	0.94 (0.91 to 0.96)
65–74 years	1.09 (1.07 to 1.12)
75–84 years	2.04 (2.00 to 2.09)
≥85 years	4.17 (4.07 to 4.26)

**Sex**	
Male	0.73 (0.72 to 0.74)
Female	Reference

**Deprivation level, IMD decile**	
1 (most deprived)	3.15 (3.08 to 3.21)
2	2.63 (2.57 to 2.69)
3	2.00 (1.95 to 2.04)
4	1.36 (1.33 to 1.40)
5	1.25 (1.22 to 1.28)
6	Reference
7	1.06 (1.03 to 1.09)
8	0.95 (0.92 to 0.97)
9	0.89 (0.87 to 0.91)
10 (least deprived)	0.68 (0.67 to 0.70)

**Before or after the start of COVID-19**	
Before (April to September 2019)	Reference
After (April to September 2020)	0.93 (0.92 to 0.94)

a P<*0.001 for all based on joint Wald tests. IMD = Index of Multiple Deprivation.*

In primary triage 71% of calls (*n* = 70 428) were assigned to care within 1 or 2 h, whereas in secondary triage a lower proportion of 20% (*n* = 35 250) of calls were assigned to emergency or immediate care within 2 h or less (see Supplementary Table S2).

### Associations with secondary triage outcome urgency

Male patients had 6% lower odds than female patients of having an urgent triage outcome selected (requiring care within 6 h or less). The odds of an urgent triage outcome increased with increasing age and with certain symptoms (for example, urinary symptoms, chest pain, and breathlessness), but was not associated with deprivation. There were greater odds of urgent triage outcomes on a Saturday and during the afternoon period (see Supplementary Table S3).

There was substantial variation between services and between clinicians conducting triage. The OR covering the 95% mid- range of clinicians was 55, implying that the individual conducting the call is by far the strongest predictor of an urgent outcome being selected in secondary triage (see Supplementary Table S3).

### Comparing primary and secondary triage outcome urgency

Most calls, 74% (*n* = 72 836), were downgraded from primary triage, whereas 12% (*n* = 11 596) were upgraded and 15% (*n* = 14 514) stayed at the same urgency level. Supplementary Table S4 shows the percentage of calls upgraded and downgraded from the primary triage outcome. Specifically, 92% of calls (*n* = 33 394) with a primary triage outcome of ‘care within 1 h’ were downgraded and 63% of calls (*n* = 654) with a primary triage outcome of ‘self-care care/no urgency’ were upgraded.

Figure 2 demonstrates the magnitude of change between primary and secondary triage outcomes, including upgrading/downgrading by several urgency levels in some calls. The topmost grey band represents calls that were initially triaged as ‘immediate care within 1 h’ that were subsequently downgraded by five urgency levels to ‘self-care/no urgency’ (17%, *n* = 6238/36 424) calls. Conversely, 9% (*n* = 1055/11 263) of calls initially triaged as ‘care within 24 h’ were subsequently upgraded to emergency or immediate outcomes (care within 2 h or less).

Symptoms with the highest odds of upgrade from a lower primary triage outcome related to chest pain (OR 2.68, 95% CI = 2.34 to 3.07) and breathlessness (OR 1.62, 95% CI = 1.42 to 1.85). Those with highest odds of downgrade were dizziness (OR 1.93, 95% CI = 1.68 to 2.22) and earache (OR 2.15, 95% CI = 1.90 to 2.42); reference: abdominal pain (*P*≤0.001 for all). There were greatest odds of call upgrading on Saturdays (see Supplementary Table S3).

In both upgraded and downgraded calls, there was no association with sex or deprivation. There was no association with age in upgraded calls; however, there were greater odds of downgrading in younger age groups, for example, 2–4-year-olds: OR 1.35 (95% CI = 1.25 to 1.46; reference: 45–54-year-olds; *P*<0.001) (see Supplementary Table S3).

There was variation by service, and most substantially variation by clinician. The ORs covering the 95% mid-range of clinicians were 5.15 and 4.71 for upgrading and downgrading, respectively, implying that the clinician conducting the call is the strongest predictor of whether the primary urgency is changed (see Supplementary Table S3).

### Variation in triage outcomes associated with clinician

Table 2 summarises the mean number of calls triaged by clinician and the percentages/interquartile range of calls: a) with an urgent secondary triage outcome selected; b) upgraded; and c) downgraded, by clinician.

**Table 2.** Summary of urgent triage outcomes selected and upgrading/downgrading of primary triage outcome by clinician

**By clinician**	**Mean**	**Median**	**IQR**	**Variation associated with clinician (OR)^a^**
**Digital triage dataset (*n*: calls = 177 786, clinicians = 259)**				
Calls, *n*	755	378	60–1144	N/A
Urgent triage outcome selected, %	52.84	55.33	37.62–70.0	54.92

**Dataset containing primary triage urgency code (*n*: calls = 98 946, clinicians = 253)**				
Calls, *n*	1051	922	486–1382	N/A
Calls upgraded, %	11.7	10.7	8.1–14.1	5.15
Calls downgraded, %	73.6	74.5	69.0–78.8	4.71

a *OR covering 95% mid-range of clinicians. For example, clinicians with the greatest odds of selecting an urgent triage outcome, at the top of the 95% mid-range, had 55 times greater odds of selecting an urgent outcome compared with the clinicians with lowest odds to generating this, at the bottom of the 95% mid-range. IQR = interquartile range.*

*N/A = not applicable. OR = odds ratio.*

## DISCUSSION

### Summary

This is the first study, to the authors’ knowledge, to describe factors influencing secondary triage outcome urgency and the upgrading/downgrading of the primary triage urgency within England’s urgent care system. This study found report call rates to be highest in the youngest and oldest age groups, with greater call rates in female patients and in the most deprived groups. Calls about chest pain, breathlessness, and urinary symptoms had the highest odds of an urgent secondary triage outcome and had greatest odds of being upgraded from the primary triage urgency level. Although the majority (74%) of calls were downgraded from primary triage, 12% of calls were upgraded, with calls about chest pain and breathlessness having the highest odds of upgrade. There were major shifts across several urgency levels in both upgraded and downgraded calls.

Most substantially, variation in both selection of urgent secondary triage outcomes and to a lesser, but still substantial, extent the upgrading/downgrading of primary triage outcomes was associated with the individual clinician conducting triage, despite all clinicians using the same digital triage system.

### Strengths and limitations

To the authors' knowledge, this is the first large-scale, in-depth evaluation of two- step triage. The large degree of upgrading and downgrading from primary triage suggests that non-clinician triage alone cannot be relied on, emphasising the importance of clinician triage within this model of care. However, without further investigation of patients’ subsequent service use and health outcomes (for example, attendance at an emergency department or admission to hospital), it is not possible to draw definitive conclusions about the safety and reliability of this two- step model.

There are strengths and weaknesses associated with the use of routine data. Strengths include the inclusion of patient groups typically underserved in research,^18^ particularly deprived groups and older adults; however, ethnicity data were not available, which may have led to the current study missing important service-use observations.

As routine data are not collected for research purposes,^19^ variables may be limited in the information they provide. For example, the number of calls triaged within the dataset was a proxy measure for the clinician’s familiarity with digital triage; information about clinician characteristics that may have an impact on how they triage (for example, their clinical experience or background) was not available. Additionally, presenting symptom was based on the clinician’s selection of triage question; this does not provide the full picture of what was discussed during the triage call, including the patient’s description of symptoms and the advice given to the patient about the action to take if their condition worsens.

Binary indicators of call upgrading and downgrading were used in regression modelling; exploring the degree of upgrade/downgrade is important to further develop this research.

This dataset was limited to the triage outcomes derived from the software in use. This is both a strength and a limitation. On the one hand, the primary triage was all undertaken using the NHS Pathways digital triage system and the secondary triage undertaken by clinicians using the Odyssey digital triage system, so allowing rigorous comparison between the two levels of triage. However, a limitation is that the authors do not know the extent to which the differences observed between primary and secondary triage are specific to services using the Odyssey software or would apply more generically to other triage solutions. Research is needed between comparative models of secondary triage to understand the extent to which the digital triage software in use affects outcomes.

### Comparison with existing literature

Previous studies have focused on direct clinician triage,^7^^,^^12^^,^^13^ and hence represent a more diverse patient population than included here; the current study population has a lower likelihood of referral to emergency care or self-care, as such patients are unlikely to have been referred to an urgent care provider for secondary triage from primary triage.

Previous studies have suggested non- clinician triage may increase emergency/urgent care workload.^20^^–^^22^ Despite the current similar finding that primary triage is risk averse, and hence may lead to a high proportion of calls being assessed as needing urgent care, the current study additionally highlights an important safety issue: some potentially life-threatening calls appear to have clinical risk underestimated in primary triage and some are upgraded by several levels of urgency in secondary triage. This builds on findings from a recent study that linked NHS 111 calls with subsequent emergency department attendance; they reported some mis-triage, including patients who received low-urgency advice who were subsequently assessed as urgent in an emergency department, of which a proportion were admitted to hospital.^23^

Previous studies of non-digitally supported triage in urgent care have reported several factors that influence clinicians’ decision making, including their experience, confidence, and attitudes to risk, as well as service availability,^24^ the clinician’s sex,^25^ and attachment to a general practice.^26^ Although these may help explain some of the variation seen in the current study, it demonstrates the persistence of substantial variation even with clinicians using a standard digital triage system.

### Implications for research and practice

The high level of variation in how clinicians triage calls implies a need for improved clinician training, auditing, and professional development. Cooperation between providers of primary and secondary triage is also important, particularly in addressing potential safety implications of under-triage in primary triage (for example, in the calls making up the 2% of secondary triage calls referred to emergency care).

Further research is needed to better understand why and how clinician variation occurs, including the impact of service-level factors (for example, workload pressures and availability of medical personnel), clinician-related factors (for example, experience and average time taken per triage call). Patient outcomes (for example, emergency department attendance/admission to hospital following triage) should also be considered in evaluating the quality and safety of urgent care triage.

Given the workforce challenges facing health care, future research should evaluate the impact of two-step triage on clinician workload, which may be useful to countries considering change to optimise their urgent care delivery model.

In conclusion, this study highlights limitations of non-clinician primary triage and emphasises the importance of secondary triage within England’s urgent care model. Although primary triage is risk averse by design, this study suggests underestimation of clinical risk in certain calls that are subsequently upgraded, with some being triaged to emergency/immediate care. Greatest odds of this occurred in calls about chest pain and breathlessness, and calls made on Saturdays, highlighting the need to specifically look at ways of reducing the clinical risk associated with such presentations during triage. Additionally, there is unexplained inconsistency between clinicians’ secondary triage, despite all using the same digital triage system. Further research is needed to address these areas of risk, to ensure patients receive urgent care in a timely fashion according to their clinical needs.
